# Various Patterns of Composition and Accumulation of Steroids and Triterpenoids in Cuticular Waxes from Screened Ericaceae and Caprifoliaceae Berries during Fruit Development

**DOI:** 10.3390/molecules24213826

**Published:** 2019-10-23

**Authors:** Soyol Dashbaldan, Rafał Becker, Cezary Pączkowski, Anna Szakiel

**Affiliations:** 1 Department of Plant Biochemistry, Faculty of Biology, University of Warsaw, 1 Miecznikowa Street, 00-927 Warsaw, Poland; dsoyloo89@gmail.com (S.D.); r.becker@biol.uw.edu.pl (R.B.); myhacp@biol.uw.edu.pl (C.P.); 2 Mongolian University of Science and Technology, School of Industrial Technology, 8nd khoroo, Baga toiruu 34, Sukhbaatar district, Ulaanbaatar 14191, Mongolia

**Keywords:** cuticular waxes, fruit development, GC-MS, sterols, triterpenoids

## Abstract

Cuticular waxes are primarily composed of two classes of lipids: compounds derived from very-long-chain fatty acids and isoprenoids, particularly triterpenoids and steroids. Isoprenoids can occur in cuticular waxes in high amounts, dominating the mixture of aliphatic long-chain hydrocarbons, while in other plants they are found in trace concentrations. Triterpenoids occurring in fruit cuticular waxes are of interest due to their potential role in the protection against biotic stresses, including pathogen infections, and their impact on the mechanical toughness of the fruit surface, maintaining fruit integrity, and post-harvest quality. The aim of the present study was the determination of the changes in the triterpenoid profile of the fruit cuticular waxes of four plant species bearing edible berries: *Vaccinium myrtillus*, *V. vitis-idaea*, and *Arbutus unedo* of the Ericaceae and the edible honeysuckle *Lonicera caerulea* of the Caprifoliaceae. Triterpenoids were identified and quantified by GC-MS/FID (gas chromatography-mass spectrometry/flame ionization detection) at three different phenological stages: young berries, berries at the onset of ripening, and mature berries. During fruit development and maturation, the triterpenoid content in cuticular waxes displayed species-specific patterns of changes. The steroid content seemed to be directly correlated with the developmental stage, with a very typical point of transition between growth and ripening being observed in all the fruit analyzed in this study.

## 1. Introduction

The aerial surfaces of the primary parts (leaves, flower petals, fruit, and non-woody stems) of all terrestrial plants are covered with a hydrophobic layer called a cuticle [1,2]. The main physiological function of the cuticle is to prevent desiccation of the plant organs due to uncontrolled non-stomatal water loss, as well as the loss of organic and inorganic compounds by leaching. However, the cuticle also serves as the first protective barrier against abiotic and biotic environmental stresses and the first contact zone in the interaction of plants with other organisms at all trophic levels [3]. Specifically, the cuticle is designed to protect the plant surfaces against mechanical damage, abrasions, infection by pathogens, insect herbivore attack, infiltration of xenobiotics, and potentially harmful irradiance, such as UV-B radiation [1,4].

The functionally important lipophilic fraction of the cuticle consists of a structural matrix called cutin and waxes. Cutin is a polyester-type biopolymer, composed mainly of hydroxy- and hydroxyepoxy-fatty acids. Waxes are embedded in the cutin and form a continuous layer above it, so that intracuticular and epicuticular wax layers can be distinguished [5,6]. Waxes can take diverse forms, from amorphous to crystalline deposits, and consist of complex mixtures of long-chain aliphatic and cyclic components, including primary alcohols, hydrocarbons, esters, fatty acids, as well as triterpenoids and steroids—plant isoprenoids synthesized from isopentenyl diphosphate via their common C30 precursor, the long-chain hydrocarbon squalene [7,8].

The triterpenoid content of cuticular waxes has been long considered to be uninteresting and poor by comparison to the profile of these compounds occurring in entire organs [9]. The development of suitable analytical methods revealed that this opinion could be caused by the predominance (mainly in fruit cuticular waxes) of one compound, usually a triterpene acid, e.g., ursolic acid in apples or oleanolic acid in grapes [8,10]. The high abundance of this prevailing compound often masked the other triterpenoids present in much lesser amounts. According to current data [11,12,13], the chemical composition of cuticular waxes shows great variability, not only among different plant species, but also between different organs of an individual plant, and is affected by the stage of plant development.

The occurrence of nonsterol triterpenoids (very often pentacyclic) in cuticular waxes is widely known; nevertheless, the presence of steroids (phytosterols and steroid ketones) in plant surface layers requires special attention. Their occurrence and function in surface waxes are particularly interesting, since these compounds are regarded as primary metabolites participating in the structure and fluidity regulation of cellular membranes rather than as passive constituents of the plant surface layer. Cuticular steroids include commonly occurring phytosterols, such as sitosterol, stigmasterol, and campesterol, sometimes accompanied by their respective stanols; however, compounds such as cholesterol, various derivatives of cycloartenol, as well as steroid ketones (e.g., stigmasta-3,5-dien-7-one) are also often found in plant cuticles. Cuticular pentacyclic triterpenoids include acids and neutral compounds (alcohols, aldehydes, and ketones) with various carbon skeletons (oleanane, ursane, and lupane-type skeletons occur widely, but friedooleanane, friedoursane, taraxastane, taraxerane, and others are also found, depending on the plant species). The results of qualitative and quantitative GC-MS analyses of the triterpenoids occurring in the fruit and leaf cuticular waxes of higher plants belonging to different taxonomic families pointed to the existence of several characteristic patterns of distribution of these compounds, e.g., the accumulation of high amounts of triterpenoids (mainly the prevailing pentacyclic acids) in the fruit wax, usually accompanied by their lower levels in the leaves, as opposed to low amounts of triterpenoids, often with predominating steroids, more abundant in the leaves than in the fruit. Among the plants studied so far, good examples of the first strategy are ericaceous plants (e.g., bilberry *Vaccinium myrtillus* or heather *Calluna vulgaris*), which additionally exhibit a particular diversity of triterpenoid skeletons [11,12]. In turn, steroids were found to predominate in both the fruit and leaf cuticles of the edible honeysuckle, *Lonicera caerulea* [14]. The total content of triterpenoids in cuticular waxes varies substantially among species (e.g., from only 6.4% of the wax extract mass in honeysuckle berries to nearly 80% in young *Vitis vinifera* grapes) [10,14]. Triterpenoids are often more abundant in fruit than in leaf cuticles (up to 10-fold higher in the fruit wax than in the leaf wax of *V. vinifera*, almost twice as much in the bilberry, *V. myrtillus*) [10,11]; however, this rule is not general (the honeysuckle again being an exception among the analyzed plant species [14]).

The presence of triterpenoids in fruit cuticles is of interest due to their potential role in protection against abiotic and biotic stresses, modulation of the mechanical toughness of the fruit peel, surface texture, appearance, and the post-harvest quality of fruit [8,13,15]. The process of fruit ripening involves many chemical changes, both in the mesocarp (e.g., the breakdown of storage polysaccharides, degradation of acids, and the transformation of pectins into water-soluble forms) as well as in the outermost layer, the epicarp. Nevertheless, the processes occurring in the cuticular wax layer on the fruit surface have not been studied extensively. The evolution of the triterpenoid content during fruit ripening can be important for understanding the progressive changes in susceptibility to pathogens as well as the mechanical properties of the fruit surface. The possible compositional changes in triterpenoids can also be of interest in terms of the metabolic pathways of these compounds in the fruit epidermis and their subsequent transport to cuticular waxes.

It was previously demonstrated that the composition of cuticular waxes in grapes (*V. vinifera*) was altered during fruit development, with an initially very high level of triterpenoids (particularly oleanolic acid) followed by a gradual decrease in this level until full ripening [10]. Such a pattern of triterpenoid alteration has also been demonstrated for some other fruit, e.g., sweet cherry (*Prunus avium*) fruit [16]; however, it is not a general rule in fruit ontogeny. In contrast, triterpenoids were shown to be progressively accumulated during olive fruit development, and the profile of these compounds was altered according to the different phases in their long maturation [17]. These observations have revealed that the changes occurring in triterpenoid accumulation in cuticular waxes during fruit development are not easily predictable, and that such studies require case-by-case investigation. As concluded by Lara et al. (2015), a survey on the compositional evolution of specific cuticular components during fruit development indicated that any temptation to simplify or generalize this phenomenon should be ruled out, and any generalization should be treated with caution [13].

The aim of the present study was the determination of the changes in the triterpenoid profile of fruit cuticular waxes of four plant species bearing edible berries, including three plants from the Ericaceae family, i.e., *V. myrtillus*, *V. vitis-idaea*, and *Arbutus unedo*, and one plant from the Caprifoliaceae family, the edible honeysuckle *L. caerulea*. The triterpenoid content was determined by a GC-MS method at three different phenological stages: young berries, berries at the onset of ripening, and mature berries. The obtained results enhance our knowledge of the diversity and distribution of triterpenoids occurring in fruit cuticular waxes and our understanding of the compositional and functional evolution of cuticular waxes during fruit development.

## 2. Results and Discussion

### 2.1. Lingonberry (Vaccinium vitis-idaea L., Family Ericaceae)

**Figure 1:**
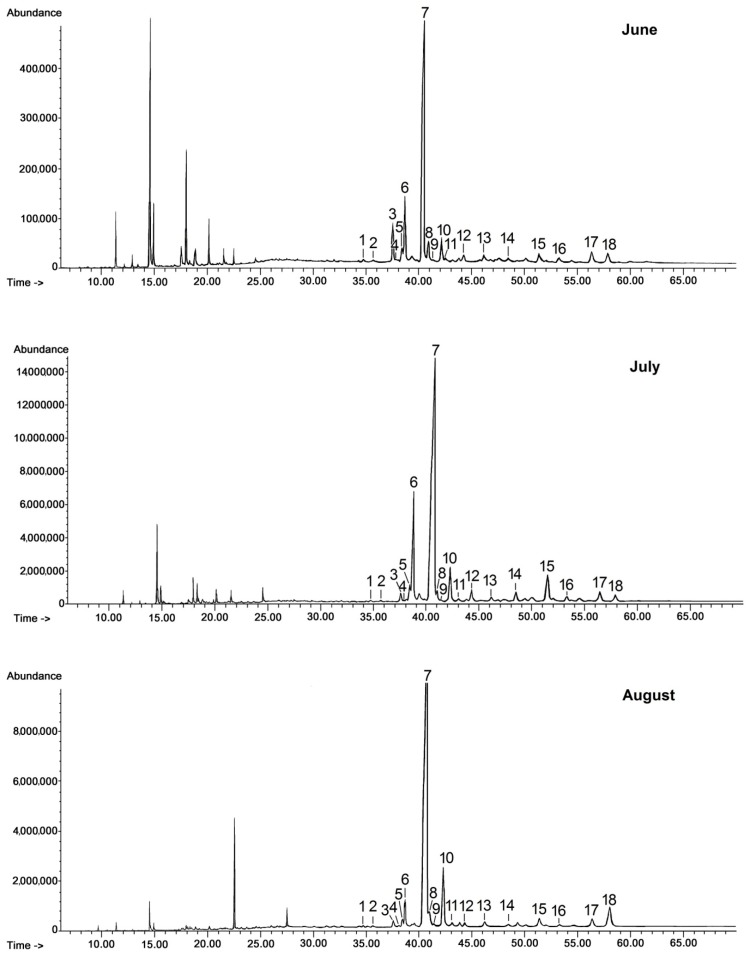
GC-FID chromatograms of the fraction of steroids and neutral triterpenoids obtained from chloroform extracts of cuticular waxes of lingonberry fruit harvested in three developmental stages (young berries in June, unripen full-size berries in July, and ripen berries in late August). 1—campesterol, 2—stigmasterol, 3—sitosterol, 4—sitostanol, 5—cycloartanol, 6—β-amyrin, 7—α-amyrin/lupeol, 8—tremulone, 9—fernenol, 10—24-methylenecycloartanol, 11—svertenol, 12—taraxasterol, 13—friedelin, 14—oleanolic aldehyde, 15—ursolic aldehyde, 16—erythrodiol, 17—uvaol, 18—betulin.

Chloroform extracts of cuticular waxes were fractionated by preparative TLC (thin layer chromatography) as described in Section 3.3. The representative GC chromatograms of fractions containing free steroids and neutral triterpenoids (alcohols, ketones, and aldehydes) are presented in Figure 1.

The triterpenoid profile of the lingonberry fruit wax appeared to be very complex. It consisted of pentacyclic compounds of ursane-, oleanane-, lupane-, fernane-, friedelane-, swertane-, and taraxerane-type carbon skeletons, including several monohydroxy alcohols: α- and β-amyrins, fern-7-en-3β-ol, lupeol, swert-9(11)-en-3β-ol, and taraxasterol, three dihydroxy alcohols: erythrodiol, uvaol, and betulin, one ketone: friedelin, two aldehydes: oleanolic and ursolic, and their corresponding acids: oleanolic and ursolic. Two identified compounds, i.e., α-amyrin and lupeol, formed one common peak, which had been observed in a previous study for diethyl ether extracts obtained from dried entire lingonberry fruit collected in 2010 [18]. The identification of these compounds in the mixture was confirmed by GC-MS analysis of their authentic standards, examined separately or combined. Apart from the pentacyclic compounds, several steroids were also identified, including the most typical phytosterols (campesterol, stigmasterol, and sitosterol), the saturated form of sitosterol—sitostanol, one steroidal ketone—tremulone (stigmasta-3,5-dien-7-one), and precursors of sterols (the saturated form of cycloartenol, i.e., cycloartanol, and its derivative, 24-methylenecycloartanol).

**Figure 2:**
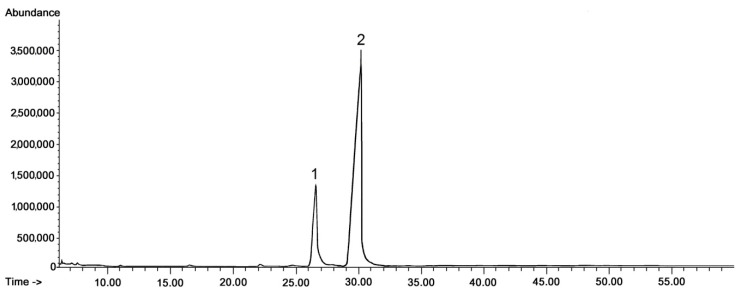
GC-FID chromatograms of the fraction of methylated triterpenoid acids obtained from chloroform extracts of cuticular waxes of lingonberry fruit harvested in July. Methyl esters of: 1—oleanolic acid, 2—ursolic acid.

The general composition of the fraction of steroids and neutral triterpenoids stayed constant during the tested period of fruit development; however, some changes in ratio among individual compounds could be expected on the basis of the visible differences in some peak heights (and thus their areas applied for quantification of the content). In the methylated triterpenoid acid fraction only two compounds were identified, i.e., the methyl esters of oleanolic and ursolic acid, with the ratio between these two compounds not significantly changing on chromatograms obtained for different fruit developmental stages. Therefore, only one representative chromatogram of such a fraction is presented in Figure 2.

The current identification of triterpenoids found in lingonberry fruit is generally consistent with the results of earlier analyses [18]. However, the identification of triterpenoids occurring in chloroform extracts from cuticular waxes, as observed for other plants, e.g., bilberry *V. myrtillus* [11], seems to be easier and more precise than analyses of triterpenoids in diethyl ether extracts obtained from the respective entire plant organs, due to the lack of contamination with various low-polarity phenolics and products of chlorophyll degradation. In the case of lingonberry, this more precise analysis permitted oleanolic and ursolic aldehydes to be identified, as well as sitostanol and 24-methylenecycloartanol, which were not found in the previous study on entire fruit.

The results of the quantification of individual compounds (made using an external standard method based on the FID signal, as described in Section 3.6) are presented in Table 1. Due to the coelution of α-amyrin and lupeol forming one common peak, their amounts were counted together. It should be noticed that the used method of wax extraction does not give the 100% yield that is necessary for absolute quantification of extracted compounds. However, despite potential incomplete recovery (up to 73% of the cuticular wax, depending on the plant species [19]), the brief, gentle method of chloroform extraction of surface waxes is commonly applied because it precludes solvent penetration across the cuticle and prevents contamination by compounds originating from deeper tissues, such as the epidermis, collenchyma, or parenchyma [8]. Moreover, despite this drawback, such studies fully enable the comparative evaluation of the relative changes in the content of pentacyclic triterpenes and steroids during fruit development [10].

The total content of triterpenoids was the highest in the cuticular waxes of the young fruit, and it accounted for more than 70% of the chloroform wax extract. It decreased to 55% of the wax extract during the intensive fruit growth phase, and afterward it again increased a little (to 60%) in fully ripe fruit. Triterpenoid acids were the predominant compounds in lingonberry fruit wax at all developmental stages. In the young fruit their content accounted for 83% of all triterpenoids in the wax, reaching 603 mg/g of wax extract (i.e., more than 60% of the wax extract mass). During the following steps of fruit ontogeny, the content of acids progressively decreased to less than 42% of the wax extract mass, constituting 75 and 69% of all triterpenoids in green full-size berries and ripe berries, respectively. Ursolic acid was the most abundant triterpenoid in lingonberry fruit wax, significantly prevailing over its isomer, oleanolic acid. The ratio of ursolic to oleanolic acid did not change markedly during fruit development: in the young fruit it equaled 4.6:1, and then it slightly decreased to approx. 4:1.

The content of the neutral pentacyclic triterpenoid fraction (mono- and dihydroxy alcohols, aldehydes, and ketones) in the waxes of lingonberry fruit was significantly lower than that of the triterpenoid acids, starting from 8% of the wax extract mass in the young fruit and reaching 12% in the ripe fruit. The amount of these compounds showed a constant trend of steady increase during fruit development, and finally, in the ripe fruit, it rose to a level higher by 50% than that found in young fruit. The most prominent compounds of this fraction were the mixture of α-amyrin with lupeol followed by fernenol.

The content of steroids in the cuticular wax of lingonberry fruit was relatively low, ranging from approx. 2 to 3.6% of the total wax extract mass. It displayed a different trend of accumulation than the pentacyclic neutral triterpenoids. It decreased by 34% from the stage of the young green berries to the stage of the fully developed but unripe fruit. It then increased markedly (by 45%) during ripening to a level higher by almost 20% than in the initial stage of the young fruit. Sitosterol was the most abundant compound in this fraction.

The content of the triterpenoid ester fraction was the lowest, not accounting even for 3% of the total wax extract mass. It increased by a factor of two during fruit development. Sitosterol esters were dominant among the compounds of this fraction, followed by esters of α-amyrin, fernenol, and ursolic acid.

Summarizing, the total content of triterpenoids in the cuticular waxes of lingonberry fruit was the highest in the young berries and the lowest in fully developed but unripe berries. During the final stage of ripening the level of triterpenoids slightly increased; however, it was still much below the initial level of these compounds detected in the young, still-growing berries (Figure S1).

This considerable decrease in the total level of triterpenoids during lingonberry fruit development seems to be parallel to the trend observed in the content of triterpenoid acids. Obviously, it is unlikely that ursolic and oleanolic acids, once accumulated in cuticular waxes, could be either transported back to the cell or undergo additional chemical transformations (e.g., degradation or disintegration). Hence, this decrease in triterpenoids could rather be explained by a simultaneous increase in the level of the aliphatic constituents of cuticular waxes and, consequently, by the dilution of triterpenoid acids in the lingonberry wax extract mass. It can be assumed that with the growing size of the berry, and the increase in water content during fruit ripening, some aliphatic hydrophobic compounds (primarily hydrocarbons) are progressively accumulated in increased amounts in the surface layers of the fruit cuticle to prevent the uncontrolled loss of water, simultaneously diluting the initial high concentration of triterpenoid acids in waxes.

The decrease in the level of triterpenoid acids points to a progressive reduction in their rate of biosynthesis. Simultaneously, the accumulation of neutral pentacyclic triterpenoid compounds, including the precursors of these acids (amyrins), increased. Thus, either the biosynthesis of triterpenoid acids is reduced after the formation of amyrins, or their accumulation in lingonberry cuticular waxes is stopped during fruit maturation, and they are redirected to other locations in fruit, for example the seeds. Nevertheless, the increasing accumulation of neutral pentacyclic triterpenoids in cuticular waxes cannot fully compensate for the decrease in triterpenoid acid deposition, thus leading to the final decrease in total triterpenoid content.

### 2.2. Bilberry (Vaccinium myrtillus L., Family Ericaceae)

The profile of the neutral triterpenoids identified in bilberry fruit cuticular wax appeared to be simpler than that described previously for lingonberry. Apart from both amyrins, their respective ketones (α- and β-amyrenones) were identified. However, other compounds belonging to the ursane and oleanane series were not found in the analyzed samples, i.e., neither dihydroxy alcohols (uvaol and erythrodiol), nor ursolic and oleanolic aldehydes were identified in bilberry fruit wax. The other pentacyclic triterpenoids present, previously found in lingonberry, were lupeol and taraxasterol, and the only new identified compound was D:C-friedours-7-en-3-ol.

**Figure 3:**
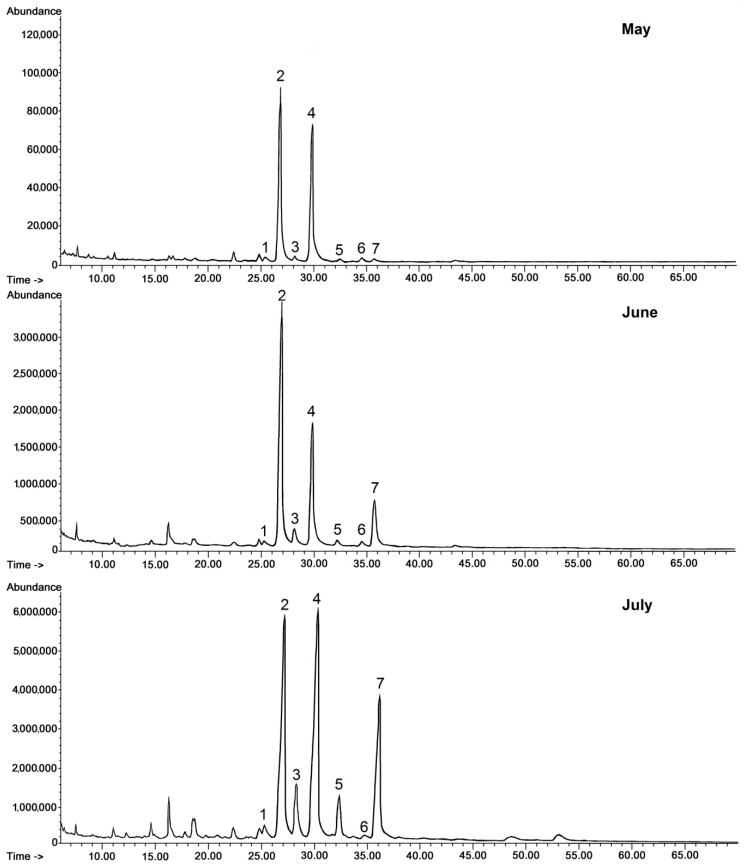
GC-FID chromatograms of the fraction of triterpenoid acids (after methylation) obtained from chloroform extracts of cuticular waxes of bilberry fruit harvested in three developmental stages (young berries in May, unripen full-size berries in mid-June, and ripen berries in early July). Methyl esters of: 1—3-oxo-oleanolic acid, 2—oleanolic acid, 3—3-oxo-ursolic acid, 4—ursolic acid, 5—maslinic acid, 6—corosolic acid, 7—oleanolic acid acetate.

Regarding steroids, the same typical composition as in lingonberry was also identified in bilberry fruit wax. In contrast, the composition of the triterpenoid acid fraction was more complex, because apart from oleanolic and ursolic acids, their 3-oxo-analogs (3-oxo-olean-12-en-28-oic and 3-oxo-urs-12-en-28-oic acids) and their dihydroxy analogs (2,3-dihydroxyoleanolic and 2,3-dihydroxyursolic acids, synonyms: maslinic and corosolic acids) were also identified, as well as the acetate ester of oleanolic acid. The abundance and the ratio of these compounds changed visibly along with fruit development, as can be clearly seen on the presented chromatograms (Figure 3.)

The profile of triterpenoid compounds occurring in bilberry fruit wax therefore consists of pentacyclic compounds of ursane-, oleanane-, lupane-, friedoursane-, and taraxerane-type carbon skeletons, including five monohydroxy alcohols: α- and β-amyrins, D:C-friedours-7-en-3-ol, lupeol, and taraxasterol, two ketones: α- and β-amyrenones, as well as two acids: oleanolic and ursolic, along with their 3-oxo- and 2,3-dihydroxy analogs, and one ester derivative, oleanolic acid acetate. The profile of steroids is identical to lingonberry, being composed of campesterol, stigmasterol, sitosterol, sitostanol, cycloartanol, 24-methylenecycloartanol, and tremulone.

The current identification of triterpenoids found in bilberry fruit is generally consistent with the previous results [11]; however, in the present study three new compounds have been found in the triterpenoid acid fraction: 3-oxo-oleanolic and 3-oxo-ursolic acids, as well as oleanolic acid acetate. Again, neither friedelin nor friedelinol (compounds occurring in bilberry fruit collected in Finland) were detected in our analyzed samples, which originated from the Mazovia region of Poland.

The results of the quantification of individual compounds are presented in Table 2. The accumulation pattern of the total content of triterpenoids in bilberry fruit cuticular wax appeared to be different than in lingonberry fruit wax. The total triterpenoid content was lowest in the cuticular waxes of the young fruit (constituting 36% of the chloroform wax extract), then it increased to more than 40% of the extract mass, and during maturation it remained at a practically similar level (approx. 39% in fully ripe fruit). However, every class of analyzed compounds displayed a different tendency of accumulation. The levels of the neutral pentacyclic triterpenoids and esters grew constantly during fruit development, whereas the level of steroids decreased significantly (by 34%) during the young fruit growth, and afterward increased again during maturation, almost reaching the initial level.

As in the lingonberry, triterpenoid acids were the predominant compounds in bilberry fruit wax at all developmental stages. In the young fruit their content accounted for 71% of all triterpenoids in the wax, then – during fruit growth – it increased to 78% (316 mg/g of wax extract, i.e., almost 32% of the wax extract mass), and finally decreased during fruit maturation to 70% of all triterpenoids (approx. 28% of the wax extract mass). The most abundant triterpenoid was oleanolic acid, followed by its isomer, ursolic acid. The amount of oleanolic acid was not significantly higher than ursolic acid, the ratio of oleanolic to ursolic acid in young and mature fruit equaling approx. 1.1:1. Only in fully grown but immature fruit collected in June was this ratio a little higher, reaching almost 1.3:1.

A very characteristic phenomenon observed during bilberry fruit development was the sharp increase in the content of oleanolic acid acetate. The amount of this ester rose 12-fold in growing fruit, and then doubled during maturation, thus increasing 22.5-fold during the entire process of fruit development. This sharp enlargement of oleanolic acid acetate biosynthesis and accumulation in waxes can explain another observed phenomenon – the predominance of 3-oxo-ursolic acid over 3-oxo-oleanolic acid, which was rather unexpected according to the general trend of oleanane-skeleton dominance in the bilberry triterpenoid profile. It can be concluded that the reactions leading to oleanolic and ursolic acid derivatives occur with different rates: free oleanolic acid seems to be readily esterified to acetate ester, whereas ursolic acid is oxidized to its 3-oxo-derivative.

The content of the neutral pentacyclic triterpenoid fraction (mono- and dihydroxy alcohols and ketones) in the waxes of bilberry fruit was almost 10-times lower than that of the triterpenoid acids, showing a constant trend of slight increase during the fruit development, starting from less than 3% of the wax extract mass in the young fruit and reaching approx. 4% in the ripe fruit. The most prominent compounds of this fraction were β-amyrin and the mixture of α-amyrin with lupeol; however, β-amyrin slightly prevailed, thus confirming the slight supremacy of compounds of the oleanane-type skeleton over the ursane-type skeleton in bilberries.

The content of steroids in the cuticular wax of bilberry fruit was higher than that of neutral triterpenoids, ranging from approx. 4.7 to 7% of the total wax extract mass. As observed previously in lingonberry, the content of steroids displayed a characteristic pattern of accumulation with an initial relatively high level (approx. 7.2% of the total wax extract), which decreased by 34% from the stage of the young green berries to the stage of the fully developed but unripe fruit, and increased again to the initial level. Sitosterol was the most abundant compound in this fraction.

The content of the ester fraction was the lowest, reaching approx. 0.7% of the total wax extract mass in fully mature fruit. It increased by a factor of three during fruit development. Sitosterol esters were dominant among the compounds of this fraction, followed by the esters of oleanolic and ursolic acids.

Summarizing, the total content of triterpenoids in the cuticular waxes of bilberry fruit was lowest in the young berries and highest in fully developed but unripe berries; however, during the final stage of ripening the level of triterpenoids decreased only slightly (Figure S2).

### 2.3. Strawberry Tree (Arbutus unedo L., Family Ericaceae)

The composition and the ratio of free steroids and neutral triterpenoids, as well as that of triterpenoid acids, did not change spectacularly during strawberry fruit development. The profile of the neutral triterpenoids identified in the cuticular wax of strawberry tree fruit contained the typical triterpenoids with ursane- and oleanane-type skeletons (α- and β-amyrins, α- and β-amyrenones, dihydroxy alcohols: uvaol and erythrodiol, aldehydes: ursolic and oleanolic), as well as the lupane-type mono- and dihydroxy alcohols, lupeol, and betulin. However, other compounds were also identified in this fraction, including taraxasterol, which was described earlier in both the lingonberry and bilberry, and three compounds not found before: moretenol, hopenone, and oleandione. In comparison to the pentacyclic triterpenoids, the composition of steroids identified in cuticular wax of strawberry tree fruit seemed to be rather simple, consisting of the most typical phytosterols, i.e., campesterol, sitosterol, and stigmasterol, along with cycloartanol and tremulone. In turn, the composition of the fraction of triterpenoid acids were even more complex than that for the bilberry. The assorted profile of oleanolic and ursolic acids, their 3-oxo-analogs (3-oxo-olean-12-en-28-oic and 3-oxo-urs-12-en-28-oic acids), analogs with additional double bond in position 2 (olean-2,12-dien-28-oic and ursa-2,12-dien-28-oic acids), dihydroxy analogs (2,3-dihydroxyoleanolic and 2,3-dihydroxyursolic acids, i.e., maslinic and corosolic acids) in addition to 3,19-dihydroxyoleanolic acid, i.e., pomolic acid, were identified. However, corosolic and pomolic acids were found only in cuticular waxes extracted from green berries, but they were not present in the more developed and ripe fruit.

The profile of triterpenoid compounds occurring in cuticular wax of strawberry tree fruit consists of pentacyclic compounds of ursane-, oleanane-, lupane-, taraxerane- and hopane-type carbon skeletons, including five monohydroxy alcohols: α- and β-amyrins, lupeol, moretenol, and taraxasterol; three ketones: α- and β-amyrenones, and hopenone; one diketone: oleandione; two dihydroxy alcohols: erthrodiol and uvaol; as well as two acids: oleanolic and ursolic, along with their various derivatives: 3-oxo-, 2,12-dien-, 2,3-dihydroxy- and 3,19-dihydroxy analogs. The profile of steroids is simpler than in the lingonberry and bilberry, being composed only of campesterol, stigmasterol, sitosterol, cycloartanol, and tremulone. The results obtained in this study generally confirmed the results obtained earlier for the entire strawberry tree fruit [20]; however, as in the case of bilberry, the analysis performed for cuticular waxes permitted to identify more compounds occurring in minor amounts, e.g., moretenol and hopenone, not found before.

The results of the quantification of individual compounds are presented in Table 3. The general accumulation pattern of the total content of triterpenoids in the cuticular wax of the strawberry tree fruit differed from the pattern described for the ericaceous berries characterized previously. The total triterpenoid content was the highest in the cuticular waxes of the young green fruit (constituting 66% of the chloroform wax extract), and it decreased progressively to 63% in yellow fruit and 56% in fully ripe red fruit. Neutral triterpenoids and acids displayed the same decreasing tendency of accumulation. Only the level of esters grew constantly during fruit development, whereas the level of steroids displayed the accumulation pattern observed previously for lingonberry and bilberry, i.e., it decreased (by 20%) during the young fruit growth, and afterward increased again during maturation, reaching a level of 24% higher in red fruit than the initial level in green fruit.

In contrast to both fruit described before, the predominating fraction in strawberry tree fruit cuticular waxes at all developmental stages was that of the neutral triterpenoids. Their content was relatively stable during fruit development, constituting from 67% to 76% of all the triterpenoids in the wax of green and red fruit, respectively (more than 40% of the wax extract mass at every fruit developmental stage). The most prominent compounds were α-amyrin, lupeol, and β-amyrin, followed by taraxasterol. The triterpenoid acid fraction was less than half that of the neutral triterpenoids, constituting 30% of all the triterpenoids in the cuticular waxes of the green fruit, but decreasing by almost half in red fruit waxes. Ursolic acid predominated among the acids, its level accounting for 80% of this fraction, and it was more than 5-times higher than that of oleanolic acid, with the ratio of ursolic to oleanolic acid being 5.2:1 in green fruit, and even 5.4:1 in red fruit. Some acids present in relatively low amounts in the waxes of green fruit, i.e., 3-oxo-oleanolic, corosolic, and pomolic acids were not found in detectable amounts in the waxes of ripening fruit. In turn, derivatives with additional double bonds (olean-2,12-dien-28-oic and ursa-2,12-dien-28-oic acids) appeared during fruit maturation, in yellow and red fruit.

The content of steroids in the cuticular wax of strawberry tree fruit was very low, ranging from approx. 2% to 3.5% of the total wax extract mass. Sitosterol was the most abundant compound in this fraction, and the only sterol detected in the ester fraction. Other compounds identified in ester fraction were the prevailing triterpenoids of the two dominating fractions, i.e., amyrins, taraxasterol, and oleanolic and ursolic acids (Figure S3).

### 2.4. Edible Honeysuckle (Lonicera caerulea L., Family Caprifoliaceae)

The profile of the pentacyclic triterpenoids was the poorest among all the plants analyzed in this study. It consisted only of five compounds of oleanane-, ursane-, and hopane-type skeleton, i.e., α- and β-amyrins, oleanolic and ursolic acids, and hopenone. The profile of steroids was a little more complex, including campesterol, sitosterol, stigmasterol, but also cholesterol, cycloartanol and its hydroxyl derivative, cycloartenediol.

The quantification of individual compounds is presented in Table 4. The total content of triterpenoids and steroids in the cuticular waxes of honeysuckle fruit was found to be extremely low in comparison to ericaceous berries, with the highest level not exceeding 6% of the total wax extract mass in green developing fruit, and the lowest amount being 3.6% in the fully developed but unripe fruit. In contrast to the previously observed profiles, steroids were the prevailing compounds in the honeysuckle fruit cuticular wax, constituting 76 and 71% of the total triterpenoid content in young and mature berries, respectively, and decreasing to 50% in fully developed unripe berries. Sitosterol dominated in this fraction, constituting more than 50% of the total steroid content at every developmental stage. The accumulation pattern of the pentacyclic neutral triterpenoids and triterpenoid acids was different, with the highest level reached at the stage of fully developed unripe berries. The content of neutral triterpenoids increased by 30% during fruit growth, but during subsequent maturation it declined even below the initial level in young berries. Similarly, the content of triterpenoid acids increased by 12% during fruit expansion and decreased by 21% during ripening, to a level 10% lower than in the young fruit.

The neutral pentacyclic triterpenoid fraction, with hopenone as the most prominent constituent, was over 5-times more abundant than the acid fraction. Ursolic acid prevailed over oleanolic acid, with the ratio of ursolic to oleanolic acid being 3.9:1 in ripe berries. The ester fraction was composed only of sterols, and their accumulation pattern was parallel to the tendency exerted by the free steroid fraction, with a decrease at the stage of fully developed berries, but with a subsequent 2-fold increase in the ripe fruit (Figure S4).

**Figure 4:**
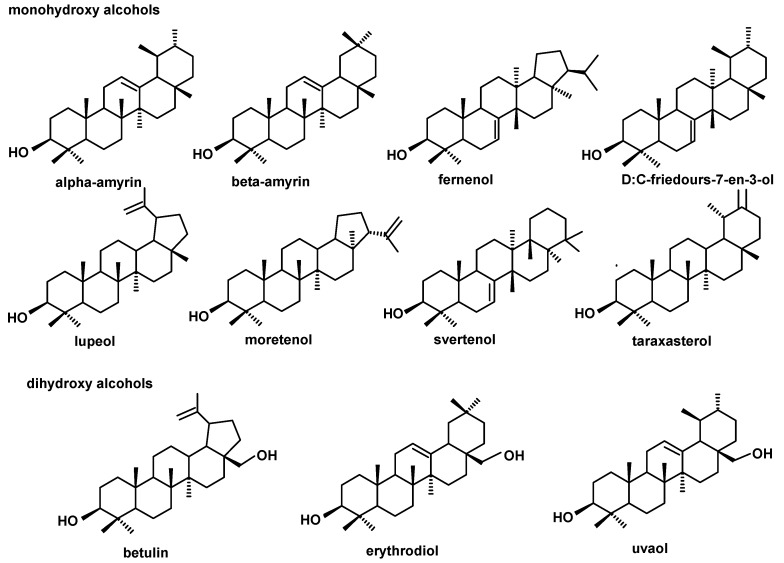
The structures of pentacyclic neutral triterpenoids (mono- and dihydroxy alcohols) identified in cuticular waxes of fruit analyzed in this study.

**Figure 5:**
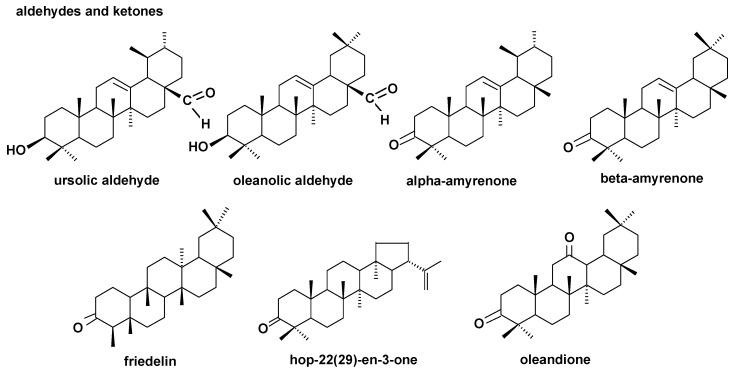
The structures of pentacyclic neutral triterpenoids (aldehydes and ketones).

**Figure 6:**
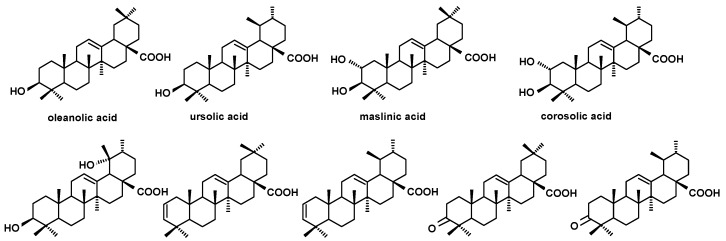
The structures of triterpenoid acids identified in cuticular waxes of fruit analyzed in this study.

**Figure 7:**
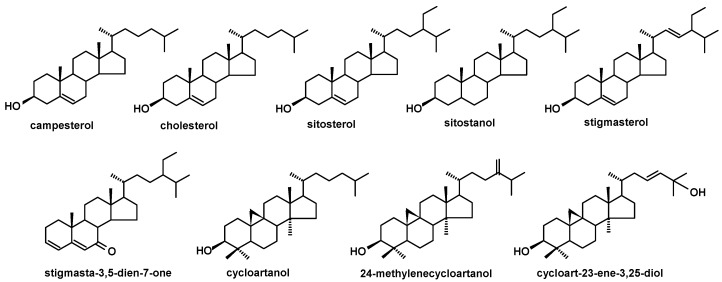
The structures of steroids identified in cuticular waxes of fruit analyzed in this study.

The structures of all the steroids and triterpenoids identified in this study are presented in Figure 4, Figure 5, Figure 6 and Figure 7. Characteristical ions of their mass spectra are presented in Table S1.

## 3. Material and Methods

### 3.1. Plant Material

Lingonberry *Vaccinium vitis-idaea* L. and bilberry *Vaccinium myrtillus* L. (Ericaceae) fruit were collected from natural forest habitat in the central Mazovia region of Poland (52°455 N, 21°332 E). Voucher specimens were deposited in the herbarium of the University of Warsaw (accession no. WA 0000017596 and WA 0000027907 for lingonberry and bilberry, respectively). Fruit of the edible honeysuckle cultivar *Lonicera caerulea* L. (Caprifoliaceae) var. *kamtschatica* “Chernichka” (Czerniczka) and strawberry tree *Arbutus unedo* L. (Ericaceae) were collected from a private plantation in Stare Bosewo, central Poland (52°460 N, 21°332 E). Saplings of *L. caerulea* were purchased from the Polish vegetable seed farming and nursery enterprise “PNOS” and cultivated in an open field. Saplings of *A. unedo* were purchased from the Polish plant nursery “World Deciduous Plants” and cultivated in a greenhouse with winter heating (this plant, native to the Mediterranean region, is not fully frost-tolerant at this latitude, and it does not survive outdoors in winter).

All fruit were collected at three different stages: young berries, fully developed but unripe berries, and mature berries. To avoid the impact of plant inter-individual variability, the harvested samples of lingonberry and bilberry originated from the same ramets (i.e., individuals originating vegetatively from a single ancestor and connected by rhizomes, thus regarded as genetically identical), whereas the samples of edible honeysuckle and strawberry tree were collected at every developmental stage from the same plants. The replicates of approx. 2 g samples of berries were prepared from pooled sample sets of 10–15 g.

### 3.2. Chemicals and Standards

All solvents: chloroform, diethyl ether, methanol (purchased from Chempur, Piekary Śląskie, Poland) used for extraction and analysis were of analytical grade. Authentic standards of α-amyrin and ursolic acid methyl ester were purchased from Roth (Karlsruhe, Germany); β-amyrin, lupeol, uvaol, oleanolic acid, campesterol, sitosterol and stigmasterol were purchased from Sigma-Aldrich (Steinheim, Germany). α-Amyrenone and β-amyrenone were obtained by oxidation of the α-amyrin and β-amyrin standards with chromium trioxide-pyridine in dichloromethane [18].

### 3.3. Extraction and Fractionation

#### 3.3.1. Extraction of Waxes

The procedure of wax extractions was reported previously [10,11,12]. Each sample of entire berries was extracted by incubation in appropriate volume of chloroform (approx. 10-times more than the volume of extracted plant material) with gentle stirring for 30 s at room temperature. All experiments were done in three replicates. The extracts were decanted, filtered, evaporated to dryness under a gentle stream of nitrogen and weighted.

#### 3.3.2. Fractionation of Chloroform Extracts

Evaporated chloroform extracts were fractionated by adsorption preparative TLC on 20 cm × 20 cm glass plates coated manually with silica gel 60H (Merck, Darmstad, Germany). The solvent system chloroform:methanol 97:3 (*v*/*v*) was applied for developing. Three fractions were obtained as described earlier: free (non-esterified) steroids and triterpenoids, triterpenoid acids, and steroid/triterpenoid esters [10]. Fractions were eluted from the gel in diethyl ether. Subsequently, fractions containing free neutral triterpenes and sterols (*R*_F_ 0.3–0.9) were directly analyzed by GC-MS, fractions containing triterpene acids (*R*_F_ 0.2–0.3) were methylated with diazomethane, and fractions containing triterpenoid (triterpene and sterol) esters (*R*_F_ 0.9–1) were subjected to alkaline hydrolysis. The average recovery of α-amyrin, uvaol, stigmasterol, and ursolic acid methyl ester from preparative TLC plates was 98.6, 97.2, 98.9 and 96.1%, respectively.

### 3.4. Derivatization of Triterpenoid Acids

Nitrosomethylurea (2.06 g) was added to a mixture of 20 mL of diethyl ether and 6 mL of 50% aqueous potassium hydroxide and the organic layer was then separated from the aqueous layer. Samples containing triterpenoid acids were dissolved in 2 mL of the obtained solution of diazomethane in diethyl ether, and held at 2 °C for 24 h.

### 3.5. Alkaline Hydrolysis

The ester fraction was subjected to alkaline hydrolysis with 10% sodium hydroxide in 80% MeOH at 80 °C for 3 h. Subsequently, 5 volumes of water were added to each hydrolysate, the pH was neutralized with 5% CH_3_COOH, and the obtained mixtures were extracted with diethyl ether (3 × 10 mL). These extracts were fractionated by preparative TLC as described above, then fractions containing free triterpene alcohols and sterols were directly analyzed by GC-MS, while triterpene acid fractions were methylated prior to this analysis.

### 3.6. Identification and Quantification of Steroids and Triterpenoids by GC-MS/FID

An Agilent Technologies 7890A gas chromatograph (Perlan Technologies, Warszawa, Poland) equipped with a 5975C mass spectrum detector was used for qualitative and quantitative analyses. Samples dissolved in diethyl ether:methanol (5:1, *v*/*v*) were applied (in a volume of 1–4 μL) using 1:10 split injection. The column used was a 30 m × 0.25 mm i.d., 0.25-μm, HP-5MS UI (Agilent Technologies, Santa Clara, CA, USA). Helium was used as the carrier gas at a flow rate of 1 mL/min. The separation was made either under isothermal conditions at 280 °C or with the following temperature program: initial temperature of 160 °C held for 2 min, then increased to 280 °C at 5 °C/min, and the final temperature of 280 °C held for a further 44 min. The other employed parameters were as follows: inlet and FID (flame ionization detector, part of 7890A chromatograph) temperature 290 °C; MS transfer line temperature 275 °C; quadrupole temperature 150 °C; ion source temperature 230 °C; EI 70 eV; *m/z* range 33–500; FID gas (H_2_) flow 30 mL·min^−1^ (hydrogen generator HydroGen PH300, Peak Scientific, Inchinnan, UK); and air flow 400 mL·min^−1^. Individual compounds were identified by comparing their mass spectra with spectral libraries (Wiley 9th ED. and NIST 2008 Lib. SW Version 2010), previously reported data, and the results of earlier experiments, as well as by comparison of their retention times and corresponding mass spectra with those of authentic standards, where available. Quantitation of was conducted with an FID detector and performed using an external standard method based on calibration curves determined for authentic standards of ursolic acid methyl ester, α-amyrin and stigmasterol [11].

### 3.7. Statistical Analysis of Data

All experiments were performed in triplicate. Data are presented as the means ± standard deviation of three independent samples analyzed in triplicate. The data were subjected to one-way analysis of variance (ANOVA), and the differences between means were evaluated using Duncan’s multiple-range test. Statistical significance was considered to be obtained at *p* < 0.05.

## 4. Conclusions

The results obtained in this study show that the triterpenoid content in fruit waxes displays considerable variability among species and confirm the inappropriateness of attempting generalizations on the composition of fruit cuticular waxes. However, some similarities among phylogenetically close species could be observed. The surface waxes of fruit of the family Ericaceae, known for the high level of triterpenoids accumulated in free forms, were generally rich in these compounds; however, the initial high level generally decreased during ripening. The ratio of triterpenoid acids vs. triterpenoid alcohols seems to distinguish the plants belonging to the Vaccinium genus from those of the Arbutus genus. In contrast, triterpenoids were not abundant in the cuticular waxes of the fruit of the plant belonging to another family, Caprifoliaceae (edible honeysuckle, *L. caerulea*); moreover, the profile of these compounds was dominated by steroids and not by pentacyclic triterpenoids.

The level of steroid content in waxes seems to be directly dependent on the current stage of fruit development; in the growing fruit these compounds are required for membrane building in multiplying cells and their level in waxes is low. The level can increase later, when the fruit reach their final size, and steroids are no longer needed for their primary function in membranes. Indeed, in all the berries analyzed in this study, at fruit developmental phases involving intensive growth by cell division and consecutive synthesis of membranes, the level of these compounds accumulated in surface waxes was relatively low. During the stages of maturation, involving mainly water and sugar accumulation, the excess sterols can be exported into cuticular waxes and accumulate there at more substantial levels.

Regarding all these observations, it can be concluded that triterpenoids seem not to be necessary components of fruit cuticular waxes. In some fruit, they occur in very high amounts, dominating the mixture of aliphatic long-chain hydrocarbons; however, in others they constitute only a minor part. Therefore, it can be hypothesized that the presence or absence of triterpenoids in cuticular waxes reflects the main chemical defense strategy of the plant and depends on the ability of the plant to synthesize them. Plants of some families, such as the Ericaceae, synthesize large amounts of triterpenoids, and hence a high level of these compounds is found in their cuticular waxes. In other families, such as the Caprifoliaceae, their chemical defense seems to be based on other types of metabolites, so triterpenoids are scarce in their cuticular waxes.

The specific composition and biochemical changes in cuticular waxes during fruit maturation and ripening are important in the determination of fruit quality attributes. Further studies are required for a better understanding of the role of steroids and triterpenoids in cuticular wax structure and particularly in the mechanical toughness of the fruit surface [21]. The results obtained in this study suggest that the high abundance of triterpenoid acids in cuticular waxes might be correlated with fruit surface durability and firmness. Previously, a molecular model of the three-dimensional arrangement of oleanolic acid and the aliphatic constituents of cuticular wax (e.g., *n*-hexacosanol) was proposed to explain such organization [22]. Two types of functional groups present in the molecule, i.e., hydroxyl and carboxyl groups, enable the formation of dimers and other possible spatial relationships with the various constituents of the cuticular wax layer. Lingonberry fruit, possessing the highest content of triterpenoid acids in their cuticular wax of the fruit analyzed in the current study, are regarded as extremely persistent, with a much longer shelf-life than the fruit of most other soft berry species, including the very fragile and easily damaged fruit of the honeysuckle and the strawberry tree. The slow spoilage of lingonberry fruit is usually explained by the presence of benzoic acid, a compound commonly used as an antimicrobial food preservative, which prevents the growth of mold and fungi. However, it cannot be ruled out that the type of fruit surface, its density, cohesiveness, toughness, and the composition of cuticular waxes also participate in the fruit’s resistance and its mechanical properties.

## Figures and Tables

**Table 1:** Content of triterpenoids in cuticular waxes during lingonberry *Vaccinium vitis-idaea* fruit development.

Compound	Content [mg/g Wax Extract ± SD (Standard Deviation)]		
	June	July	August
α-amyrin/lupeol	50.89 ± 5.25^a^	53.18 ± 5.62^a^	59.01 ± 6.40^a^
β-amyrin	6.11 ± 0.59^a^	8.28 ± 0.90^b^	10.78 ± 1.14^c^
betulin	0.37 ± 0.04^a^	1.46 ± 0.12^b^	4.66 ± 0.42^c^
erythrodiol	0.44 ± 0.05^a^	0.81 ± 0.09^b^	1.03 ± 0.11^c^
fernenol	13.26 ± 1.24^a^	15.12 ± 1.50^a^	17.44 ± 1.68^a^
friedelin	3.14 ± 0.33^a^	5.26 ± 0.48^b^	7.82 ± 0.84^c^
oleanolic aldehyde	0.34 ± 0.03^a^	1.12 ± 0.12^b^	2.48 ± 0.25^c^
svertenol	3.12 ± 0.29^a^	4.68 ± 0.42^b^	5.26 ± 0.58^b^
taraxasterol	1.48 ± 0.13^a^	2.93 ± 0.27^b^	3.18 ± 0.32^c^
ursolic aldehyde	2.08 ± 0.18^a^	4.15 ± 0.38^b^	6.24 ± 0.52^c^
uvaol	0.50 ± 0.05^a^	2.01 ± 0.23^b^	4.75 ± 0.49^c^
Sum of neutral pentacyclic triterpenoids	8.73	99.00	122.65
oleanolic acid	106.80 ± 12.62^a^	87.03 ± 8.75^b^	82.50 ± 7.98^b^
ursolic acid	496.77 ± 52.11^a^	329.15 ± 33.09^b^	331.81 ±35.73^b^
Sum of triterpenoid acids	603.57	416.18	414.31
campesterol	3.30 ± 0.34^a^	2.07 ± 0.21^b^	4.18 ± 0.44^c^
cycloartanol	1.95 ± 0.17^a^	1.08 ± 0.11^b^	2.12 ± 0.20^a^
24-methylenecycloartanol	1.84 ± 0.19^a^	0.97 ± 0.11^b^	1.76 ± 0.18^a^
sitostanol	0.87 ± 0.09^a^	0.06 ± 0.01^b^	1.04 ± 0.11^a^
sitosterol	14.55 ± 0.17^a^	11.29 ± 2.04^b^	19.01 ± 2.23^c^
stigmasterol	2.91 ± 0.32^a^	1.42 ± 0.15^b^	3.43 ± 0.37^a^
tremulone	4.03 ± 0.41^a^	2.76 ± 0.28^b^	4.54 ± 0.52^a^
Sum of steroids	29.45	19.65	36.08
esters:			
α-amyrin	2.06 ± 0.25^a^	3.72 ± 0.38^b^	5.89 ± 0.64^c^
β-amyrin	0.02 ± 0.01^a^	1.05 ± 0.22^b^	1.74 ± 0.19^c^
fernenol	3.14 ± 0.37^a^	4.07 ± 0.51^b^	5.62 ± 0.65^c^
24-methylenecycloartanol	n.d.	n.d.	0.09 ± 0.01
oleanolic acid	0.93 ± 0.11^a^	1.68 ± 0.23^b^	2.22 ± 0.20^c^
ursolic acid	3.25 ± 0.28^a^	3.94 ± 0.42^a^	4.16 ± 0.38^a^
sitosterol	6.32 ± 0.64^a^	4.57 ± 0.51^b^	8.98 ± 0.93^c^
stigmasterol	n.d.	n.d.	1.04 ± 0.01
Sum of esters	15.72	19.03	29.74
Total	730.47	553.86	602.78

Results as referenced to wax extract mass and expressed as the mean ± SD of three independent samples analyzed in triplicate. Results in rows not sharing a common letter are significantly different (*p* < 0.05).

**Table 2:** Content of triterpenoids in cuticular waxes during bilberry *Vaccinium myrtillus* fruit development.

Compound	Content [mg/g Wax Extract ± SD]		
	May	June	July
α-amyrin/lupeol	6.46 ± 0.66^a^	8.74 ± 0.88^a^	10.35 ± 0.91^a^
α-amyrenone	1.9 ± 0.10^a^	2.81 ± 0.16^b^	3.15 ± 0.30^b^
β-amyrin	7.70 ± 0.53^a^	9.64 ± 0.63^b^	10.92 ± 1.15^b^
β-amyrenone	3.92 ± 0.28^a^	4.22 ± 0.35^a^	4.71 ± 0.43^a^
friedours-7-en-3-ol	4.81 ± 0.35^a^	5.09 ± 0.44^a^	5.68 ± 0.48^a^
taraxasterol	4.80 ± 0.29^a^	4.96 ± 0.28^a^	5.12 ± 0.50^a^
Sum of neutral pentacyclic triterpenoids	29.59	35.46	39.93
oleanolic acid	131.95 ± 11.84^a^	153.90 ± 14.65^a^	105.09 ± 8.81^b^
ursolic acid	115.92 ± 10.01^a^	121.05 ± 12.82^a^	91.47±5.09^b^
3-oxo-oleanolic acid	0.95 ± 0.01^a^	3.14 ± 0.28^b^	4.85 ± 0.30^c^
3-oxo-ursolic acid	1.82 ± 0.12^a^	6.34 ±0.58^b^	14.31 ± 1.03^c^
maslinic acid	0.31 ± 0.01^a^	4.85 ± 0.41^b^	11.87 ± 0.65^c^
corosolic acid	2.47 ± 0.25^a^	2.66 ± 0.12^a^	3.34 ± 0.22^b^
oleanolic acid acetate	2.10 ± 0.18^a^	24.59 ± 1.35^b^	45.46 ± 1.66^c^
Sum of triterpenoid acids	255.52	316.53	276.39
campesterol	4.35 ± 0.39^a^	2.48 ± 0.22^b^	3.95 ± 0.38^a^
cycloartanol	4.14 ± 0.36^a^	1.37 ± 0.10^b^	3.95 ± 0.35^a^
24-methylenecycloartanol	4.06 ± 0.38^a^	2.19 ± 0.15^b^	3.84 ± 0.28^a^
sitostanol	4.08 ± 0.30^a^	2.63 ± 0.24^b^	4.78 ± 0.40^a^
sitosterol	43.12 ± 4.06^a^	28.08 ± 2.60^b^	40.83 ± 4.02^a^
stigmasterol	3.06 ± 0.28^a^	0.91 ± 0.08^b^	2.90 ± 0.23^a^
tremulone	8.38 ± 0.74^a^	8.95 ± 0.83^a^	10.16 ± 1.08^a^
Sum of steroids	71.19	46.61	70.41
esters:			
α-amyrin	0.08 ± 0.01^a^	0.24 ± 0.02^b^	0.28 ± 0.03^b^
β-amyrin	0.16 ± 0.01^a^	0.48 ± 0.05^b^	0.55 ± 0.06^b^
24-methylenecycloartanol	0.23 ± 0.02^a^	0.57 ± 0.06^b^	0.69 ± 0.06^b^
oleanolic acid	0.95 ± 0.08^a^	1.60 ± 0.15^b^	1.72 ± 0.16^b^
ursolic acid	0.70 ± 0.05^a^	1.24 ± 0.11^b^	1.3 ± 0.14^b^
sitosterol	0.99 ± 0.07^a^	1.70 ± 0.16^b^	1.95 ± 0.20^b^
stigmasterol	tr.	tr.	0.12 ± 0.01
Sum of esters	2.12	5.83	6.69
Total	358.42	404.43	393.42

Results as referenced to wax extract mass and expressed as the mean ± SD of three independent samples analyzed in triplicate. Results in rows not sharing a common letter are significantly different (*p* < 0.05).

**Table 3:** Content of triterpenoids in cuticular waxes during strawberry tree *Arbutus unedo* fruit development.

Compound	Content [mg/g Wax Extract ± SD]		
	Green	Yellow	Red
α-amyrin/lupeol	246.32 ± 26.84^a^	235.41 ± 24.10^a^	227.65 ± 30.67^a^
β-amyrin	78.29 ± 6.61^a^	76.09 ± 7.10^a^	73.58 ± 6.92^a^
α-amyrenone	3.38 ± 0.42^a^	3.84 ± 0.36^a^	4.97 ± 0.45^b^
β-amyrenone	2.75 ± 0.21^a^	3.49 ± 0.33^b^	4.30 ± 0.39^c^
betulin	0.73 ± 0.08^a^	1.07 ± 0.10^b^	1.35 ± 0.11^c^
erythrodiol	1.12 ± 0.10^a^	1.14 ± 0.12^a^	1.18 ± 0.12^a^
hopenone	12.97 ± 1.35^a^	13.58 ± 1.44^a^	13.87 ± 1.45^a^
lupeol acetate	6.90 ± 0.72^a^	6.44 ± 0.58^a^	5.86 ± 0.60^a^
moretenol	15.41 ± 1.65^a^	15.04 ± 1.48^a^	13.39 ± 1.43^a^
oleandione	1.02 ± 0.10^a^	0.90 ± 0.08^a^	0.76 ± 0.08^a^
oleanolic aldehyde	3.62 ± 0.34^a^	3.88 ± 0.42^a^	4.35 ± 0.45^a^
taraxasterol	51.85 ± 4.80^a^	56.34 ± 5.26^a^	58.05 ± 6.01^a^
ursolic aldehyde	10.65 ± 1.09^a^	11.47 ± 1.11^a^	11.86 ± 1.20^a^
uvaol	2.74 ± 0.26^a^	3.16 ± 0.32^a^	3.77 ± 0.40^a^
Sum of neutral pentacyclic triterpenoids	438.90	431.85	424.94
oleanolic acid	30.81 ± 2.95	26.79 ± 2.50	11.54 ± 2.02
ursolic acid	157.15 ± 17.01	141.96 ± 16.06	87.52 ± 8.22
olean-2,12-dien-28-oic acid	n.d.	0.32 ± 0.01	0.13 ± 0.01
ursa-2,12-dien-28-oic acid	n.d.	1.33 ± 0.11	0.61 ± 0.05
3-oxo-oleanolic acid	0.58 ± 0.01	0.31 ± 0.01	n.d.
3-oxo-ursolic acid	1.78 ± 0.18	0.83 ± 0.09	0.62 ± 0.05
maslinic acid	1.85 ± 0.20	0.91 ± 0.10	0.17 ± 0.01
corosolic acid	0.74 ± 0.08	n.d.	n.d.
pomolic acid	3.76 ± 0.34	n.d.	n.d.
Sum of triterpenoid acids	196.67	173.18	100.59
campesterol	0.91 ± 0.10^a^	0.76 ± 0.08^a^	1.43 ± 0.11^b^
cycloartanol	1.15 ± 0.11^a^	0.73 ± 0.07^b^	1.37 ± 0.12^a^
sitosterol	9.99 ± 0.89^a^	7.86 ± 0.82^b^	13.58 ± 0.14^c^
stigmasterol	0.19 ± 0.02^a^	0.17 ± 0.02^a^	0.50 ± 0.04^cb^
tremulone	2.45 ± 0.21^a^	2.19 ± 0.20^a^	2.47 ± 0.23^a^
Sum of steroids	14.69	11.71	19.35
esters:			
α-amyrin	4.76 ± 0.52^a^	5.20 ± 0.48^a^	5.92 ± 0.62^a^
β-amyrin	0.81 ± 0.08^a^	1.08 ± 0.10^a^	1.12 ± 0.10^a^
taraxasterol	0.64 ± 0.07^a^	0.79 ± 0.08^a^	0.86 ± 0.09^a^
oleanolic acid	0.54 ± 0.06^a^	0.65 ± 0.06^a^	0.78 ± 0.08^a^
ursolic acid	0.52 ± 0.05^a^	0.83 ± 0.08^b^	1.04 ± 0.10^b^
sitosterol	0.95 ± 0.10^a^	0.88 ± 0.09^a^	1.23 ± 0.12^b^
Sum of esters	8.22	9.43	11.05
Total	658.48	626.17	555.93

Results as referenced to wax extract mass and expressed as the mean ± SD of three independent samples analyzed in triplicate. Results in rows not sharing a common letter are significantly different (*p* < 0.05).

**Table 4:** Content of triterpenoids in cuticular waxes during edible honeysuckle *Lonicera caerulea* fruit development.

Compound	Content [mg/g Wax Extract ± SD]		
	April	May	June
α-amyrin	3.39 ± 0.41^a^	4.92 ± 0.54^b^	3.48 ± 0.36^a^
β-amyrin	2.05 ± 0.19^a^	3.16 ± 0.32^b^	2.19 ± 0.23^a^
hopenone	5.17 ± 0.48^a^	6.18 ± 0.63^a^	4.67 ± 0.51^a^
Sum of neutral pentacyclic triterpenoids	10.61	14.26	10.34
oleanolic acid	0.45 ± 0.05^a^	0.54 ± 0.04^a^	0.38 ± 0.03^a^
ursolic acid	1.62 ± 0.17^a^	1.83 ± 0.20^a^	1.49 ± 0.12^a^
Sum of triterpenoid acids	2.07	2.37	1.82
campesterol	3.58 ± 0.41^a^	1.16 ± 0.10^b^	2.45 ± 0.21^c^
cholesterol	4.34 ± 0.39^a^	2.87 ± 0.31^b^	4.68 ± 0.49^a^
cycloartanol	3.17 ± 0.33^a^	1.52 ± 0.16^b^	3.04 ± 0.32^a^
cycloartenediol	1.14 ± 0.10^a^	tr.	0.89 ± 0.09^a^
sitosterol	25.53 ± 3.05^a^	9.18 ± 1.26^b^	23.15 ± 3.03^a^
stigmasterol	1.87 ± 0.27^a^	0.46 ± 0.05^b^	1.16 ± 0.12^c^
tremulone	4.72 ± 0.56^a^	2.91 ± 0.35^b^	4.82 ± 0.56^a^
Sum of steroids	44.35	18.10	40.19
esters:			
cholesterol	0.32 ± 0.04^a^	0.33 ± 0.03^a^	0.76 ± 0.09^b^
sitosterol	0.84 ± 0.11^a^	0.79 ± 0.09^a^	1.83 ± 0.22^b^
stigmasterol	0.57 ± 0.07^a^	0.52 ± 0.06^a^	1.09 ± 0.11^b^
Sum of esters	1.73	1.64	3.68
Total	58.76	36.37	56.08

Results as referenced to wax extract mass and expressed as the mean ± SD of three independent samples analyzed in triplicate. Results in rows not sharing a common letter are significantly different (*p* < 0.05).

## Supplementary Materials

Table S1: Retention times and characteristic ions of mass spectra of identified steroids and triterpenoids. Figure S1: Changes in the content of triterpenoids in cuticular waxes during lingonberry *Vaccinium vitis-idaea* fruit development. Figure S2: Changes in the content of triterpenoids in cuticular waxes during bilberry *Vaccinium myrtillus* fruit development. Figure S3: Changes in the content of triterpenoids in cuticular waxes during strawberry tree *Arbutus unedo* fruit development. Figure S4: Changes in the content of triterpenoids in cuticular waxes during honeysuckle *Lonicera caerulea* fruit development.
