# Supplementary material for: Various Patterns of Composition and Accumulation of Steroids and Triterpenoids in Cuticular Waxes from Screened Ericaceae and Caprifoliaceae Berries during Fruit Development
Source: Molecules. 2019 Oct 23;24(21):3826. doi: 10.3390/molecules24213826 (PMC6864842; doi:10.3390/molecules24213826)
Supplement: Supplementary file 1 [file molecules-24-03826-s001.pdf]

Various Patterns of Composition and Accumulation of Steroids and Triterpenoids in Cuticular Waxes from Screened Ericaceae and Caprifoliaceae Berries during Fruit Development

Table S1. Retention times and characteristic ions of mass spectra of identified steroids and triterpenoids

Range of retention time	Compound	Mass spectrum *m/z* (relative intensity)
31.8-32.0	Cholesterol	386 (26), 107 (50), 105 (48), 91 (57), 81 (54), 79 (46), 69 (47), 57 (87), 55 (73), 43 (100), 41 (55)
34.4-34.5	Campesterol	400 (30), 107 (51), 105 (55), 95 (49), 83 (45), 81 (64), 71 (62), 57 (77), 55 (77), 43 (100), 41 (52)
35.5-35.6	Stigmasterol	412 (36), 145 (64), 107 (52), 95 (100), 83 (66), 81 (90), 78 (60), 69 (67), 67 (85), 55 (69)
37.4-37.6	Sitosterol	414 (29), 145 (54), 107 (59), 105 (60), 95 (54), 91 (49), 81 (57), 57 (68), 55 (70), 43 (100), 41 (44)
37.6-37.8	Sitostanol	416 (31), 215 (82), 109 (58), 107 (83), 95 (81), 93 (64), 81 (84), 69 (60), 57 (64), 55 (81), 43 (100)
37.9-38.0	β-Amyrenone	424 (11), 219 (18), 218 (100), 205 (13), 203 (55), 189 (14), 109 (12), 95(16), 81 (12), 69 (14), 55 (15)
38.1-38.2	Cycloartanol	428 (4), 205 (60), 109 (98), 95 (100), 93 (64), 81 (69), 69 (78), 57 (73), 55 (82), 43 (89), 41 (67)
38.6-38.8	β-Amyrin	426 (27), 219 (18), 218 (100), 203 (49), 189 (17), 135 (11), 109 (13), 105 (12), 95 (15), 81 (18), 69 (14)
40.0-40.2	α-Amyrenone	424 (12), 219 (19), 218 (100), 203 (24), 189 (16), 135 (19), 133 (18), 122 (18), 119 (17), 95 (16), 55 (18)
40.4-40.8	α-Amyrin/	426 (4), 218 (100), 203 (20), 189 (36), 135 (35), 121 (32), 109 (32), 107 34), 95 (40), 81 (33), 55 (31)
	Lupeol	426 (18), 207 (67), 189 (90), 135 (83), 121 (80) 109 (85), 121 (80), 95 (100), 93 (87), 81 (86),
40.9-41.1	Tremulone (stigmasta-3,5-dien-7-one)	410 (32), 187 (27), 174 (100), 161 (37), 159 (26), 91 (28), 57 (28), 55 (37), 43 (44), 41 (28)
41.3-41.5	Fern-7-en-3β-ol	426 (12), 411 (72), 259 (100), 241 (54), 137 (54), 109 (58), 107 (62), 95 (93), 81 (77), 55 (80)
41.8-42.0	24-methylenecycloartanol	440 (5), 121 (60), 119 (55), 109 (62), 107 (76), 105 (57), 95 (98), 93 (64), 81 (72), 69 (99), 55 (100)
42.3-42.5	Svert-9(11)-en-3β-ol	426 (27), 411 (82), 393 (29), 259 (100), 241 (63), 137 (30), 119 (32), 95 (45), 81 (28), 69 (31)
42.7-42.8	Moretenol	426 (15), 207 (42), 189 (100), 147 (18), 135 (24), 107 (25), 95 (40), 81 (35), 67 (22), 55 (23)
43.5-43.7	D:C-friedours-7-en-3-ol	426 (7), 247 (100), 229 (78), 123 (49), 109 (54), 107 (48), 105 (48), 95 (77), 81 (55), 69 (66), 55 (59)

44.3-44.5	Taraxasterol	426 (14), 207 (57), 189 (100), 135 (51), 121 (74), 109 (57), 107 (62), 95 (70), 93 (47), 81 (48), 67 (43)
46.2-46.4	Friedelin	426 (6), 125 (65), 123 (78), 109 (82), 107 (46), 96 (62), 95 (94), 81 (77), 69 (100), 67 (56), 55 (71)
48.3-48.5	Oleanolic aldehyde	440 (2), 232 (28), 207 (20), 204 (39), 203 (100), 189 (29), 105 (18), 81 (19), 69 (20), 55 (29)
48.9-49.1	Cycloart-23-ene-3,25-diol	442 (10), 203 (48), 121 (73), 109 (100), 107 (82), 95 (75), 81 (91), 69 (54), 55 (62), 43 (77)
49.8-50.0	Hopenone	424 (33), 205 (29), 189 (100), 107 (39), 95 (56), 93 (29), 81 (35), 69 (36), 55 (27), 41 (21)
51.4-51.6	Ursolic aldehyde	440 (1), 207 (26), 204 (23), 203 (100), 133 (42), 119 (18), 105 (18), 95 (18), 81 (18), 55 (18), 43 (20)
53.2-53.4	Erythrodiol	442 (1), 204 (17), 203 (100), 133 (7), 119 (9), 105 (8), 95 (9), 93 (8), 81 (8), 69 (9), 55 (8)
53.9-54.1	3,12-oleandione	440 (25), 234 (92), 205 (98), 177 (98), 135 (73), 95 (100), 81 (60), 69 (72), 55 (89), 41 (55),
56.3-56.5	Uvaol	442 (1), 207 (13), 204 (17), 203 (100), 133 (33), 119 (13), 105 (11), 95 (12), 81 (10), 69 (10), 55 (11)
57.8-58.0	Betulin	442 (8), 203 (100), 189 (77), 133 (66), 121 (55), 107 (57), 105 (49), 95 (56), 93 (54), 81 (67)
Acids*:		
22.4-22.6	Olean-2,12-dien-28-oic acid methyl ester	452(11), 425 (9), 263 (11), 262 (61), 221 (14), 203 (100), 190 (15), 189 (22), 133 (14), 119 (12)
25.1-25.3	Ursa-2,12-dien-28-oic acid methyl ester	452 (12), 425 (9), 263 (20), 262 (100),221 (27), 203 (79), 190 (18), 189 (27), 133 (58), 119 (23)
25.5-25.6	3-Oxo-olean-12-en-28-oic acid methyl ester	468 (6), 262 (32), 204 (17), 203 (100), 202 (21), 189 (29),133 (17), 119 (14), 105 (12), 55 (12)
26.9-27.2	Oleanolic acid methyl ester	470 (1), 262 (48), 207 (13), 204 (16), 203 (100), 202 (21), 189 (22), 133 (17), 119 (13), 105 (14)
28.0-28.2	3-Oxo-urs-12-en-28-oic acid methyl ester	468 (3), 263 (21), 262 (96), 249 (20), 204 (17), 203 (100), 189 (29), 133 (79), 119 (30), 105 (19)
29.8-30.2	Ursolic acid methyl ester	470 (1), 263 (20), 262 (100), 207 (32), 203 (93), 189 (29), 133 (76), 119 (34), 105 (21), 95 (18)
32.0-32.2	Maslinic acid methyl ester	486 (2), 263 (10), 262 (53), 204 (17), 203 (100), 202 (20), 189 (20), 133 (16), 119 (13), 105 (12), 69 (10)
34.6-34.8	Corosolic acid methyl ester	486 (1), 263 (15), 262 (74), 204 (17), 203 (100), 202 (22), 189 (21), 119 (18), 105 (14), 55 (12)
43.0-43.4	Pomolic acid methyl ester	486 (3) 263 (12), 263 (55), 204 (16), 203 (100), 202 (20), 189 (17), 119 (12), 105 (10), 75 (14)

*retention times of metyl esters analyzed under isothermal conditions

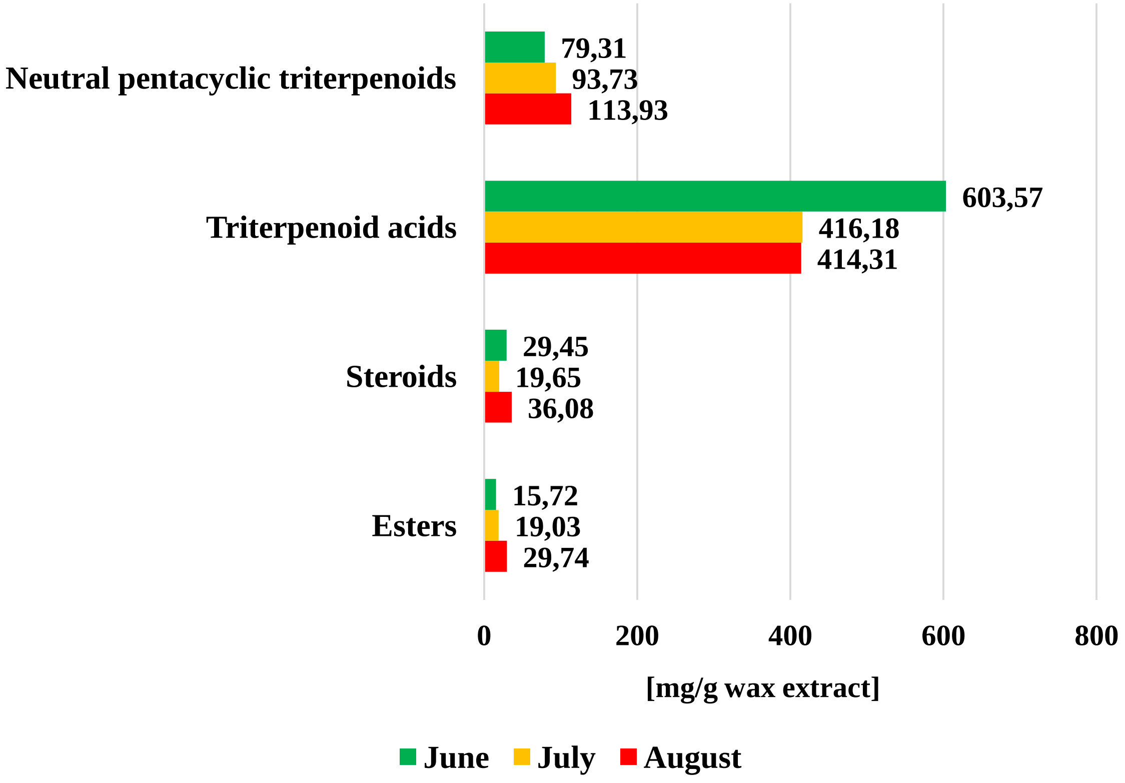

Figure S1. Changes in the content of triterpenoids in cuticular waxes during lingonberry *Vaccinium vitis-idaea* fruit development.

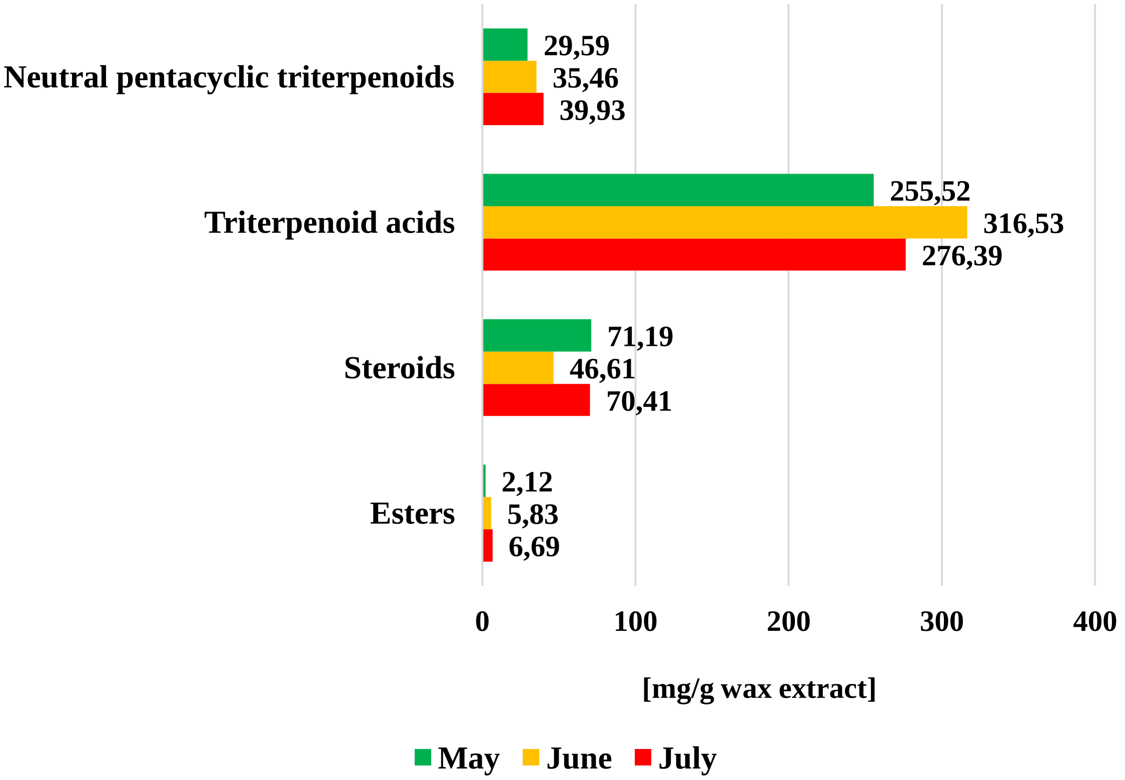

Figure S2. Changes in the content of triterpenoids in cuticular waxes during bilberry *Vaccinium myrtillus* fruit development.

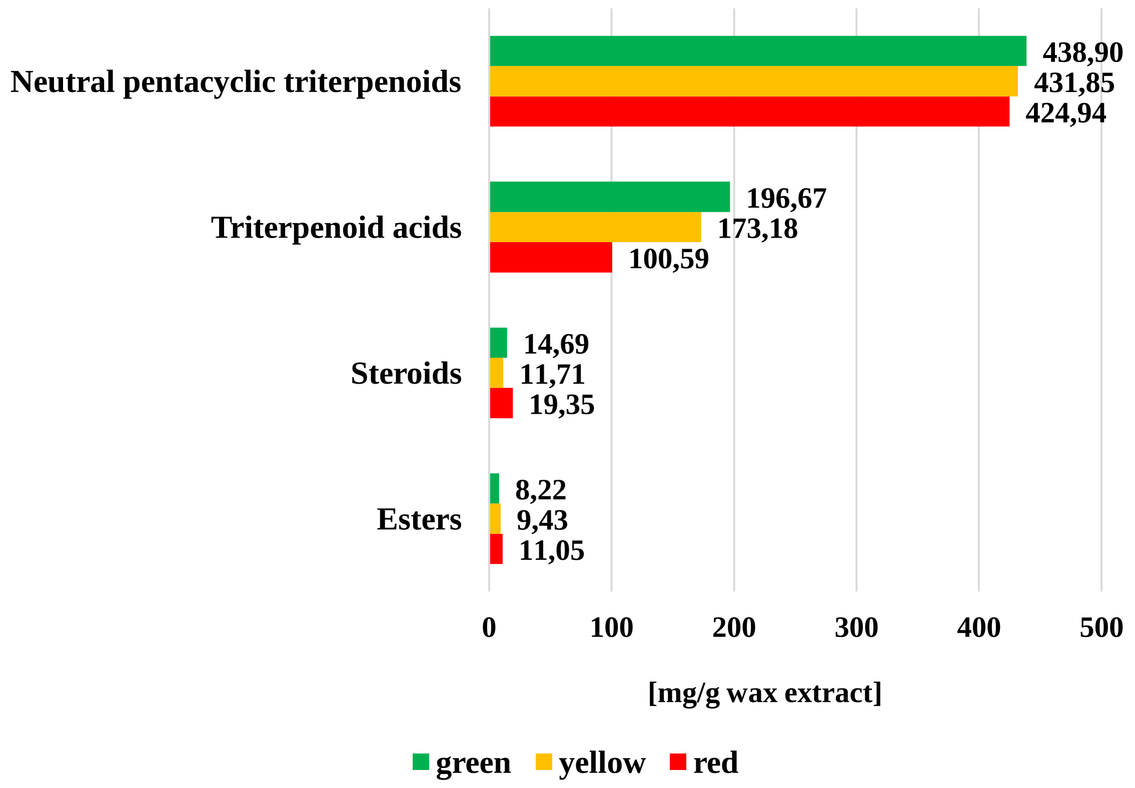

Figure S3. Changes in the content of triterpenoids in cuticular waxes during strawberry tree *Arbutus unedo* fruit development.

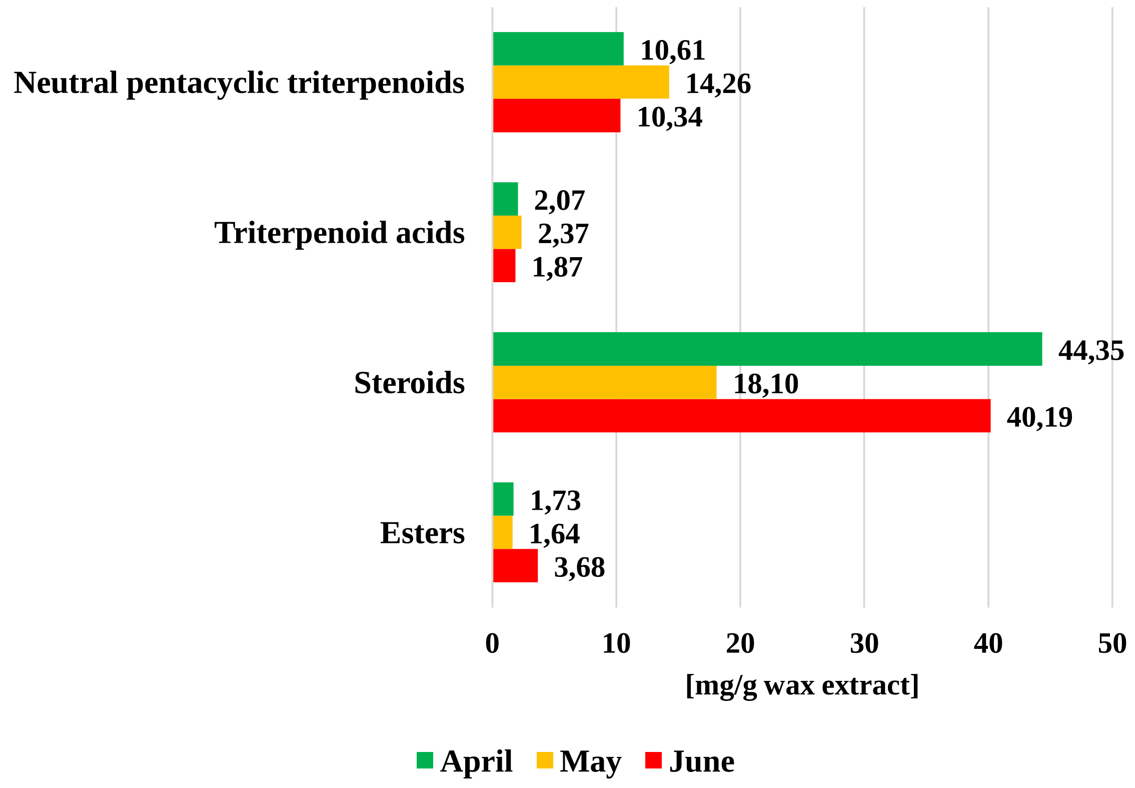

Figure S4. Changes in the content of triterpenoids in cuticular waxes during honeysuckle *Lonicera caerulea* fruit development.
